# Metal Complexes—A Promising Approach to Target Biofilm Associated Infections

**DOI:** 10.3390/molecules27030758

**Published:** 2022-01-24

**Authors:** Rodica Olar, Mihaela Badea, Mariana Carmen Chifiriuc

**Affiliations:** 1 Department of Inorganic Chemistry, Faculty of Chemistry, University of Bucharest, 90-92 Panduri Str., District 5, 050663 Bucharest, Romania; mihaela.badea@chimie.unibuc.ro; 2 Department of Microbiology, Faculty of Biology, University of Bucharest, 1-3 Aleea Portocalelor Str., District 5, 060101 Bucharest, Romania; 3 Romanian Academy of Scientists, 54 Spl. Independenței Str., District 5, 050085 Bucharest, Romania; 4 The Romanian Academy, 25, Calea Victoriei, Sector 1, District 1, 010071 Bucharest, Romania

**Keywords:** anti-biofilm activity, complex, extracellular polymeric substances, mechanism of action, metallic ion, microbial target

## Abstract

Microbial biofilms are represented by sessile microbial communities with modified gene expression and phenotype, adhered to a surface and embedded in a matrix of self-produced extracellular polymeric substances (EPS). Microbial biofilms can develop on both prosthetic devices and tissues, generating chronic and persistent infections that cannot be eradicated with classical organic-based antimicrobials, because of their increased tolerance to antimicrobials and the host immune system. Several complexes based mostly on 3D ions have shown promising potential for fighting biofilm-associated infections, due to their large spectrum antimicrobial and anti-biofilm activity. The literature usually reports species containing Mn(II), Ni(II), Co(II), Cu(II) or Zn(II) and a large variety of multidentate ligands with chelating properties such as antibiotics, Schiff bases, biguanides, N-based macrocyclic and fused rings derivatives. This review presents the progress in the development of such species and their anti-biofilm activity, as well as the contribution of biomaterials science to incorporate these complexes in composite platforms for reducing the negative impact of medical biofilms.

## 1. Introduction

Infectious diseases remain at the top of the list of mortality and morbidity causes, resulting from many converging factors such as global emergence and the spread of genetically encoded resistance to all currently used antibiotics, the delays in the discovery of novel antimicrobials, and complications associated with biofilm development on tissues and prosthetic devices [1,2]. Microbial biofilms represent the most frequent lifestyle of microbial cells in the natural environment, and in the case of pathogenic microorganisms, they protect them from adverse environmental conditions, representing both reservoirs and sources of disease outbreaks, especially in the case of medical devices [2].

The research in the field demonstrates that most bacteria, including the antibiotic-resistant ones, and some fungi could develop biofilms. Moreover, the formation of a mixed biofilms has been reported, such as *Candida*–streptococcal associations in the case of oral diseases [3]. These represent a microbial derived complex sessile community in which the microorganisms adhere irreversibly to an inert or living surface as well as an interface and to each other and are embedded in a matrix of self-generated extracellular polymeric substances (EPS) [4].

This microbial lifestyle is involved in the majority of chronic and hard to treat microbial infections, especially those associated with the healthcare system [5,6]. Microbial biofilms can form on living tissue, resulting in wound infections [7], endocarditis or lung infections in cystic fibrosis patients [6], as well as on medical devices such as stents, catheters, and prosthetic and dental implants [8].

Biofilm-embedded cells are generally much more tolerant to both antibiotics and the immune system in comparison to their planktonic counterparts [1,6,9]. The biofilm resistance towards antibiotics could be between 100–1000 fold higher in comparison to planktonic cells [10], and moreover, some antibiotics at sub-inhibitory concentrations can promote biofilm development [11]. This enhanced phenotypic resistance results from several mechanisms such as altered physiological state, the slow growth rate of bacteria, the limited penetration of antibiotics through EPS, the increased horizontal transfer of resistance genes and mutations frequency, the accumulation of antibiotic-inactivating or modifying enzymes, alterations in gene expression, and activation of quorum sensing (QS) mechanisms [12,13].

Several strategies are currently being investigated to improve the treatment of bacterial infections associated with biofilms and, moreover, to avoid the emergence of resistance and to protect the human microbiome. These strategies are based on associations of antibiotics and/or other synthetic or natural (phytochemicals, biosurfactants, antimicrobial peptides, and microbial enzymes) antimicrobial species [14,15,16], acting via interfering with the QS pathways, EPS matrix disruption, and the inhibition of microbial adhesion [17]. Moreover, a variety of bioactive substance releasing biomaterials and drug carriers have been developed to deliver antibiotics to extracellular and/or intracellular targets, or more recently, to interfere with the QS intercellular communication mechanisms. Successful controlled delivery strategies have employed dendrimers or polymeric-based formulations such as liposomes and cyclodextrins, respectively, in addition to inorganic carriers such as metal and oxide nanoparticles [6]. A thorough presentation and classification of available anti-biofilm drugs and drug delivery systems is given in many recent reviews [18,19,20].

In recent years the interest in the use of complexes for fighting biofilm associated infections has increased. These complexes exhibit multiple mechanisms of action at the microbial cell level, such as changes in microbial cell envelope permeability, reactive oxygen (ROS) or nitrogen (RNS) species release, DNA, membrane, proteins or EPS disruption, and enzyme inhibition to which an immunostimulatory effect upon the host is added [21,22,23,24,25,26,27]. These aspects assure therefore a better efficiency and a lower risk in selecting for antimicrobial resistance. Moreover, some complexes could block the QS process or inhibit the microbial adhesion [28].

The purpose of this review is to present an update of the progress in the development of the strategies based on complexes used as effective agents in biofilm associated infection prevention and treatment. After a brief description of biofilm formation and the main characteristics and strategies for its disruption, different types of complexes used to prevent or counteract these microbial multicellular forms are described. These species will be presented according to their ligand nature and mechanism of action, whenever possible, remembering that for most studies only in vitro or in vivo anti-biofilm activity are reported without significant studies for revealing the mechanism of action. Ultimately, a final discussion of challenges and future perspectives will be presented.

## 2. Aspects Concerning Biofilm Formation and Disruption

### 2.1. Biofilm Formation

Microbial biofilms developed on tissues or on the surface of implanted medical devices represent a serious and worldwide problem [8]. Some medical devices can be compromised by biofilm associated infections, such as central venous catheters, heart valves, ventricular assisting systems, coronary stents, neurosurgical ventricular shunts, implantable neurological stimulators, arthro-prostheses, fracture-fixation species, inflatable penile, mesh, breast, cochlear, joint and dental implants, as well as intra-ocular lenses [4].

The planktonic microorganisms are free-living species that float in a fluid [27]. A first step of biofilm formation is represented by microbial adhesion to an implant surface or a tissue, followed by their embedding into an EPS matrix, thus leading to an improved survival rate under a variety of stressful conditions [13]. After developing mature biofilms, planktonic bacterial cells will disperse, attaching to new surfaces and thus starting a new life cycle [28]. This growth is the result of a complex process that involves the transport of organic and inorganic molecules together with microbial cells to a living or inert surface, a subsequent adsorption to this surface, and finally an irreversible attachment assisted by EPS production [27].

**Figure 1:**
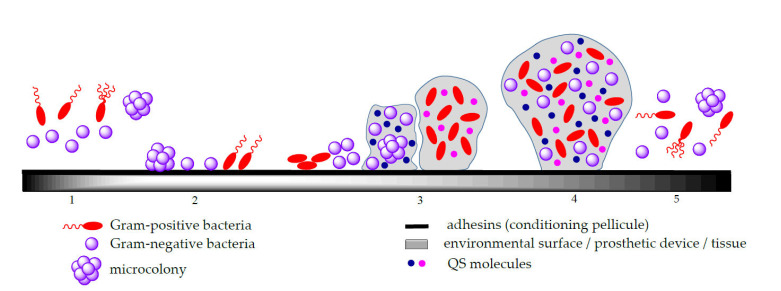
Stages in biofilm formation: 1-planktonic bacteria and microcolonies, 2-adherence, 3-multiplication and secretion of extracellular matrix, 4-mature biofilm differentiated cells and 5-dispersion of microcolonies and planktonic bacteria.

As result, biofilm development unfolds into several distinctive steps shown in Figure 1, whereas the specific mechanisms of evolution can differ based on the involved microbial populations and the local environmental conditions [29,30]. The four basic steps of biofilm development are [29,30,31,32,33,34]:

(i) Adhesion in which the planktonic cells are reversibly attached to the solid biotic/abiotic surfaces; the bacterial attachment on the biotic/abiotic surfaces involves both physical and chemical interactions such as Brownian movements, van der Waals and electrostatic attraction that contribute to the initial phase of microbial adhesion, as well as interactions between these surfaces and the bacterial adhesins, represented by polymeric species exposed on the surface of cells that enable the formation of a “key-lock” bond between the cell and the surface and result in a stronger interfacial adhesion [27,34,35,36].
(ii) Microcolony formation following initial microorganism adhesion and proliferation, with the generation of a multi-layered biofilm embedded in self-produced EPS, a complex and viscous matrix composed mainly from polysaccharides, proteins and lipids; this matrix represents the greatest barrier to diffusion for both antimicrobials and their delivery systems. In soil microbial communities, the EPS production contributes significantly to the improvement of soil quality due to its electrostatic charge that is attracting and aggregates the soil particles exerting a positive effect on the soils’ mechanical conductivity [37]. Moreover, biofilm embedded microorganisms can also produce small, diffusible signalling molecules involved in the density-dependent intercellular communication mechanism called QS; this system allows microorganisms to detect a critical density and assures a coordinated behaviour within the biofilm, such as iron chelation and antibiotic defensive activities [6].
(iii) Biofilm maturation consisting of the development of a three-dimensional structure (3D) with a thickness typically less than 100 mm and a network that assures the efficient transportation of both nutrients and signaling particles in the biofilm;
(iv) Detachment or dispersion corresponding to microcolonies or single cell separation that colonize other surfaces; after maturation, the migration of cells to the environment or dispersion is a result of a too dense layer formation [34].

Thus, there are several structural and physiological differences between planktonic and biofilm growth states, but two main distinctive factors are the presence of EPS and QS communication in the latter case.

Based on understanding the microbial biofilm formation mechanisms, traditional models of in vitro monospecific biofilm development have been developed. However, during infection, bacterial cells belonging to different species tend to form multicellular aggregates and biofilms can also disperse, not only as single cells, but also as aggregates [38]. Experimental and computational studies performed on *Pseudomonas aeruginosa* have shown that there is a competition between aggregates and single cells depending on the access to growth resources, with the balance leaning towards aggregates when competition is high. Thus, these findings show that an aggregate can outcompete the biofilm population arising from a single cell [38].

The biofilm forming abilities of microorganisms depend on several factors such as micro-environmental conditions (temperature, ionic strength and pH), the site of development, the nature of prosthetic material or tissue, nutrient type and concentration, network design and composition, strain type and heterogeneity [31].

### 2.2. Role of the Extracellular Polymeric Substances in Protecting Biofilm Cells

The biofilm gelatinous polymers known as EPS are 3D materials that carry intact microorganisms, attach them to a surface and protect them from environmental stress [32,33,34,35,36,37,38,39]. There are several types present in the environment, such as that bound to cell surfaces (“capsular” EPS), released into solution (“free” EPS), or associated with the hydrated matrix of biofilms [40]. Since EPS plays the role of a protective shelter or a diffusion barrier, the biofilms can resist external stressor attacks, such as by pH, ROS, antibiotics and phagocytosis [30,41].

This extracellular matrix is a heterogeneous mixture built from water and polymeric species, such as polysaccharides, proteins, lipids, nucleic acids and humic substances, respectively, [27,29,41,42,43] that guarantee both morphology and longevity of the biofilm [31,44].

Among these, the polysaccharides are often the most abundant species found in the biofilm matrix [45,46]. Some *Pseudomonas* EPS polysaccharides, such as the Pea and Peb, described in *P. putida*, serve structural purposes, whereas others such as alginate and cellulose play a minor role in biofilm formation and stability but are important in stress protection against ROS generated during stress [39]. The role of both alginate and cellulose in protecting against the ROS stress comes from the hydrophilic nature of polysaccharides, considering that their ability to bind water might reduce the accumulation of intracellular ROS [45].

In the EPS structure, there are several weakly acidic groups (carboxyl, phosphoryl, amide, amino, hydroxyl) that ionize in response to changes in environmental pH, ionic strength [39,40] or in interaction with a metallic ion from the environment or an antimicrobial complex. Metal-proton exchange or metal coordination is not the only mechanism involved in metal interaction with EPS, and other processes, such as cation exchange or electrostatic interactions may also occur [39,40,47]. Among the groups with coordinative abilities, hydroxyl, carboxyl, amino and phosphates represent the main sites involved in interaction with metallic ions from complexes.

Some studies indicated that both proteins and humic derivatives in EPS from activated sludge are strong ligands for Cu(II), and their carboxyl groups play an important role in Cu(II) coordination [48,49]. Furthermore, Cu(II) demonstrated a higher affinity for organic matter of biofilm in comparison with Cd(II), Zn(II) and Mn(II) [50].

### 2.3. Quorum Sensing Role in Biofilm Development

Biofilm development is closely interconnected with the QS mechanism, since its development comes from a cooperative behaviour of the microbial populations embedded in EPS. QS represents a cell-cell communication mechanism that coordinates gene expression as a response to the population cell density. Otherwise, QS synchronizes the switch to a biofilm lifestyle when the population density reaches a threshold level [4,28].

As result, biofilm maturation, its dispersion as well as virulence factors secretion are coordinated by density-dependent biochemical signals emitted in a synchronized way by the bacterial communities incorporated in EPS [28,31,51].

The extracellular signalling molecules are known as autoinducers (AIs), which are recognized by the receptors of producing and neighbouring cells. This signal is amplified and transmitted through appropriate regulatory systems, thus causing the expression of target genes [34,51]. Moreover, the signalling molecules allow bacteria to perceive and respond to temporal and contiguous environments [52].

The AIs are produced at basal level and gradually accumulate during microbial growth, creating a positive feedback loop that means that as the bacterial population grows, the concentration of the AIs in the surroundings increases, causing more inducer molecules to be synthesized [53]. The accumulation of critical concentrations of such species activates in response to the specific receptors. These are able to initiate in the biofilm a signaling cascade of coordinated induction/repression of multiple target genes, responsible for the optimal adaptation of biofilms to the biotic/abiotic media [52]. As result, QS enables microorganisms to respond quickly to environmental changes, such as the availability of nutrients, or the presence of other microbes or toxins in their environment [53].

As result, this mechanism drives both physiological and metabolic processes within a biofilm and thus controls the biofilm development. The communication differs in Gram-positive and Gram-negative bacteria, but most of the AIs are small peptides [34,52,53]. In Gram-negative bacteria, the cell-cell communication is mostly mediated by *N*-acylhomoserine lactones, alkyl quinolones and fatty acid methyl esters, diketopiperazines, furanosyl borate diester, 4,5-dihydroxy-2,3-pentanedione or 2-isocapryloyl-3*R*-hydroxymethyl-c-butyrolactone, while Gram-positive bacteria use peptides for QS activation [52,53].

The opportunistic microorganisms use the threshold AIs concentration to overcome the host defense mechanisms. To assure both infection progress and survival, microorganisms stop the synthesis of virulence factors until they reach the threshold density required for initiating the infection process [52].

### 2.4. Anti-Biofilm Strategies

The developed anti-biofilm strategies are acting at different levels:

- Inhibition of the initial microbial adhesion to the substrate, by designing materials or coatings exhibiting electrochemical repulsion or by eluting substrata loaded with antimicrobial substances [54];
- Inhibition of adhesion by blocking the expression of adhesins or their recognition sites;
- Inhibition of adhesion by blocking flagellar motility and impairing bacteria to reach the adhesion sites;
- Inhibition of biofilm formation by interfering with the QS mechanisms; the quorum quenching (QQ) by using QS inhibitors can attenuate the production of bacterial toxin or biofilm formation and provide a novel therapeutic approach to control bacterial infections. The QQ can be achieved either by blocking the QS molecules biosynthesis, by destruction of QS molecules, or by inhibition of the binding of AIs to QS receptors [55]. QSIs often exhibit a synergic anti-biofilm activity with antibiotics [56].
- Inhibition of biofilm maturation by blocking the production of extracellular polymeric substances (EPS);
- Inhibition of maturation by inhibiting the growth and multiplication of cells in biofilm by using different approaches such as application of infrared and light pulsing, direct-current electrical stimulation, ultrasound and alternating electric fields [57]; use of drug delivery systems [58]; local delivery of catheter locks, intratracheal locks etc. [59];
- Targeting non-growing dormant and persister biofilm cells (by metabolic interference or lytic substances) or interfering with the formation of persister cells (by inhibiting the bacterial regulatory tetra and penta-guanosine phosphate nucleotides, which activate the inhibitors of cell growth) [60].
- Elimination of the biofilm by disorganization of the protective extracellular matrix based on EPS-degrading enzymes, anti-EPS antibodies, and nucleic acid binding proteins, matrix destabilizing agents such as ethylenediaminetetraacetic acid (H_4_EDTA), which are targeting the biofilm extracellular polymeric substance, leading to biofilm dispersion or by the mechanical debridement of biofilms by using ultrasound and surgical procedures [61,62,63].

Although many strategies have been proposed to control biofilm development, however, very few became available to the clinicians [2]. 

## 3. Complexes with Antibiofilm Activity

One of the most promising approaches for the treatment of biofilm-associated infection is based on designing agents that exhibit multiple mechanisms of action. The special characteristic of biofilms presented above together with their resistance to classical antibiotics requires such strategies like complexes that so far demonstrated their utility, at least in vitro. 

So far, for antibiotics, the achievement of an anti-biofilm activity was reached either by modification of the conjugated moieties to the basic antimicrobial backbone or by a combinatory therapy [54,55,64]. 

By combining the special characteristic of transition metal ions with that of a proper organic scaffold, a suitable therapeutic agent can be obtained. The scientific literature reports a large diversity of species concerning both metal ions and ligands available for designing such effective anti-biofilm species ranging from simple to bulky ligands and acting as unidentate to multidentate species. A variety of complexes with anti-biofilm activity, ranging from species with known antibiotics or natural products to new synthetic ligands, mainly with low toxicity and chelating ability have been developed [65,66,67,68,69,70,71,72,73,74,75,76,77,78,79,80,81,82,83,84,85,86,87,88,89,90,91,92,93,94,95,96,97,98,99,100,101,102,103,104,105,106,107,108,109,110,111,112,113,114,115,116,117,118,119,120,121,122,123,124,125,126,127,128,129,130,131,132,133,134,135,136,137,138,139,140,141,142,143,144,145,146,147,148,149,150,151,152,153,154]. The metallic ions in these species are essential cations such Cu(I,II) [65,66,67,74,75,76,78,80,81,83,84,85,86,87,88,89,90,98,99,100,101,102,103,104,105,106,107,108,113,114,117,118,119,120,121,122,123,124,125,126,127,128,129,130,131,132,136,137,138,139,140], Co(II) [74,75,76,77,82,96,97,99,100,101,102,103,141], Mn(II,III) [68,79,93,113,114], Zn(II) [67,74,75,76,95,101,113,114,124,125,126,127,128,129,130,131,132,142,143], and Ni(II) [74,75,76,99,100,101,102,103,113,114,119,123,124,125,126,127,128,129,130,131,132,137] and those known to be less toxic, such as Pt(II) [91,119], Pd(II) [91,116,119], Au(I) [70,87], Ag(I) [69,70,85,87,91,92,94,144] and Hg(II) [69,70]. Some of the compounds thus assembled have the advantage of a positive charge and thus the ability to establish electrostatic interaction with negatively charged components of biofilm (polysaccharides, proteins and DNA), allowing them to exert an enhanced anti-biofilm activity. Also, the metal ions can extend their coordination on QS components, adhesins or the biofilm matrix, generating new mechanisms for biofilm destruction. Redox active metal ions can generate reactive inorganic molecules such as ROS or RNS that can also be involved in obtaining the desired anti-biofilm effect. In addition, the strategies based on such a compound’s incorporation in organic or inorganic carriers are currently under extensive development.

The most recent data concerning these aspects are presented in this review, with highlights on examples of complexes with antibiotics [65,66,67,68,69,70,71,72,73], N-heterocycle derivatives [74,75,76,77,78,79,80,81,82,83,84,85,86,87,88,89,90,91,92,93,94,95,96,97,98], Schiff bases [99,100,101,102,103,104,105,106,107,108], biguanides [111,112,113,114,115,116,117,118,119,120,121,122,123] and macrocycle derivatives [124,125,126,127,128,129,130,131,132] as ligands. The representative compounds for each type, together with metal ion, anti-biofilm profile and mechanism of action are provided in Table 1.

### 3.1. Complexes with Antibiotics

When the activity of antibiotics and antifungals was outclassed both by the emergence of resistance and by the reduced efficiency against biofilms, solutions were sought to overcome these problems. One of these solutions was provided by antibiotic complexation to biocations and especially to transition metal ions, which easily change their oxidation state and as a result can interact with target biomolecules involved in the destruction of the biofilm by redox processes.

Among bacteria, *Pseudomonas aeruginosa* represents an important nosocomial pathogen that is responsible for a large spectrum of infections, such as endocarditis, cystic fibrosis, burn, wound and urinary tract infections. Its pathogenicity is related to virulence factors such as biofilm formation as well as exotoxins, elastase, alginate and siderophores production [145]. The major limitation of therapy in chronic pulmonary infection is the *P. aeruginosa* biofilm formation in the lung, this being over 1000-fold more resistant to antimicrobials compared to planktonic bacteria [146]. As result, complex [Cu(Hcip)(H_2_O)_2_]SO_4_·2H_2_O (**1**) (Hcip: ciprofloxacin-a fluoroquinolone antibiotic) was studied as anti-biofilm species able to provide a high concentration of Hcip in the lungs. [65]. Besides structure determination for (**1·EtOH**), another study demonstrated that at sub-minimum inhibitory concentration (MIC), this complex exhibits a significant reduction in motility, biofilm formation, alginate, violacein and pyocyanin production and sensitivity to H_2_O_2_ in a concentration dependent manner [66]. Considering the biological effects of complex (**1**) and its inhibitory activity on QS at low concentrations, quantified through the expression of QS genes *lasI* and *lasR,* this may be used as an effective approach in the management of infections caused by this microorganism.

Complex {[ZnCl_2_(fcz)_2_]·2C_2_H_5_OH}n (**2**) (fcz: fluconazole-a triazole antifungal) showed both strong inhibition of *C. albicans* clinical isolates biofilm formation at subinhibitory concentration and the ability to reduce its adherence to human non-small cell lung cancer A549 cells in vitro. Moreover, this compound inhibits pyocyanin production and biofilm formation in *P. aeruginosa*, results that recommend its further examination in the mixed *Candida*-*P. aeruginosa* infections [67].

Compound [Mn(H_2_O)_6_]_0.5_[Mn(smx)_3_] (**3**) (smx: sulfamethoxazole–an antibiotic from the second generation of sulfonamides) was fully characterized by single crystal X-ray diffraction and proved to be an inhibitor of both the planktonic and biofilm embedded *Staphylococcus aureus* strain [68]. Complexes of Hg(II), Cu(II), Cd(II) and Ag(I) with this ligand were reported as anti-biofilm inhibitors for *E. coli* [69], while its species with Au(I), Cu(II), Ag(I), Hg(II) and Cd(II) were found to be active against biofilm produced by *Mycobacterium abscessus*, *M. fortuitum*, and *M. massiliense* strains, the most active being [Au(smx)(PPh_3_)] (**4**) (Ph: phenyl) species for all tested strains [70]. Similar activity was evidenced for Au(I) sulfadiazine complexes, an activity associated with the inhibition of cyclic-di-guanosine monophosphate (c-di-GMP) synthesis, which is an important signaling molecule for the rapidly growing mycobacteria (RGM) biofilm formation. These RGM are found in non-sterile water and are often associated with severe post-surgical infections and affect immunocompromised patients [70].

Amoxicillin (amx) is a bacteriolytic β-lactam antibiotic that inhibits the carboxypeptidase and transpeptidase required for peptidoglycan biosynthesis. Several studies revealed that its complexation is important to enhance antibacterial activity [71,72], and as a result, its species with Cu(II), Zn(II) and Fe(III) in 1:1 molar ratio were synthesized and studied against *E. coli*. The Cu(II) and Fe(III) complexes were more potent compared with Zn(II) complex with amx both on planktonic and biofilm embedded strains, the involved mechanism being oxidation by the redox active cations [73].

Hence, the coordination of fluoroquinolone, sulfonamide and β-lactam antibiotics as well as triazole antifungals to both essential and non-essential metal ions lead to an improved anti-biofilm activity of these antibiotic classes. The activity improvement could be related to both the coordinative and the redox ability of metallic ions that can interfere with either EPS or QS. The species with redox active metallic ions are by far more active as a result of ROS generation.

### 3.2. Complexes with Heterocyclic Derivatives

During the last decades, fused heterocycles bearing 1,2,4-triazolo[1,5-*a*]pyrimidine scaffold aroused the pharmaceutical interest due to their resemblance with purine bases. As result, several complexes of Co(II), Ni(II), Cu(II), Zn(II) with such ligands were developed and tested on as anti-biofilm species. Among these, the series of complexes of type [MpmtpX_2_] (pmtp: 5-phenyl-7-methyl-1,2,4-triazolo[1,5-*a*]pyrimidine; X: Cl [74], CH_3_COO [75] and ClO_4_ [76] were tested on a wide range of microorganisms such as Gram negative (*E. coli*, *K. pneumoniae*, *P. aeruginosa*) and Gram positive (*S. aureus*, *B. subtilis*) bacterial strains, as well as the *C. albicans* fungal strain. A broad spectrum of anti-biofilm activity was demonstrated by all Cu(II) species [Cu_2_(pmtp)_2_Cl_4_(OH_2_)_2_] (**5**), [Cu(pmtp)(CH_3_COO)_2_]·0.5H_2_O (**6**) and [Cu(pmtp)(OH_2_)_3_](ClO_4_)·3H_2_O (**7**) from this series at subinhibitory concentrations, including MRSA and other clinical isolates [68,69,70]. In addition, (**6**) and (**7**) also induce a decrease in the DNA content of the cells found in the G0/G1 phase for the human colon adenocarcinoma cell line (HT 29), revealing their anti-proliferative potential [75,76].

Complex bearing 5,7-dimethyl-1,2,4-triazolo[1,5-*a*]pyrimidine (dmtp) [Co(dmtp)_2_Cl_2_] (**8**) also exhibited a broad spectrum of anti-biofilm activity, being tested on the same strains [77]. Moreover, complexes with mixed ligands [Cu(bpy/phen)(dmtp)_2_(OH_2_)](ClO_4_)_2_·dmtp (**9**/**10**) proved to exhibit a stronger antimicrobial and anti-biofilm effect against the Gram-positive strains, including MRSA. In addition, compounds display an antiproliferative effect on murine melanoma (B16 cells); low toxicity on normal (BJ) cells, do not affect the membrane integrity and behave as metallonucleases [78].

The imidazole and its derivatives were also studied as ligands because of the ring presence in the protein structure as a part of histidine residues. Among these, [Mn(Him)_6_]Cl_2_·2H_2_O (**11**) (Him: imidazole) inhibits the *E. coli* biofilm [79] while [Cu_2_(acr)_4_(Hbzim/Me_2_bzim)_2_] (**12/13**) (Hacr: acrylic acid, Hbzim: benzimidazole, Me_2_bzim: 5,6-dimethylbenzimidazole) exhibited anti-biofilm activity on a wide range of bacteria (*E. coli*, *S. aureus*, *B. subtilis*, *E. faecium*) as well as *C. albicans*, activity complemented for (**12**) by an antiproliferative effect on the colon adenocarcinoma (HT29) cell line [80,81].

Good activity was reported for [Co(tcpp/tbpp)_2_Cl_2_] (**14**) (tcpp: 1-thiocarbamoyl-5-(4-chlorophenyl/)-3-phenyl-4,5-dihydro-1*H*-pyrazole; tbpp: 1-thiocarbamoyl-5-(4-bromophenyl)-3-phenyl-4,5-dihydro-1*H*-pyrazole) species on *C. glabrata* biofilm. Moreover, these complexes do not exhibit mutagenic potential or cytotoxicity against cervical cancer (HeLa, SiHa) cell lines and Vero non-tumor cells [82].

Some Cu(II) complexes of type [Cu(pym/quz)(H_2_O)_2_(NO_3_)_2_] (**15**/**16**) (pym: pyrimidine, quz: quinazoline) are able to inhibit QS by successful modulation of signalling molecules production. Aside from the inhibitory activity of complexes on the acyl homoserine and 2-alkyl-4-quinolones (AHQs) level, they are also potent inhibitors of biofilm formation in *P. aeruginosa* PAO1 [83].

Several complexes of type [Cu(cbl)(PPh_3_)_2_X] (**17**) (X: Cl, Br, I) with β-carboline (cbl) at sub-MIC concentration interfered significantly with the QS regulated functions in *Chromobacterium violaceum* (violacein), *P. aeruginosa* (elastase, pyocyanin and alginate production) and *S. marcescens* (prodigiosin). Aside from the inhibitory effect on the EPS production and swarming motility, these complexes also demonstrated potent broad-spectrum inhibition of biofilm formed by *P. aeruginosa*, *E. coli*, *C. violaceum*, *S. marcescens*, *K. pneumoniae* and *L. monocytogenes* [84]. Also, various N-heterocyclic carbene (nhc) complexes with Ag(I) and Cu(I) of type [M(nhc)Cl] (**18**) can inhibit biofilm formation of *L. monocytogenes*, *S. aureus*, *S. epidermidis*, *P. aeruginosa* and *E. coli* at low concentrations. The Ag(I) complexes of this series bearing aromatic groups on lipophilic nhc ligands exhibits the broadest anti-biofilm activity [85]. Moreover, a collection of Cu(I) complexes bearing nhc derivatives with different substituents was developed to prevent *Streptococcus mutans* biofilm formation, the most active being the less lipophilic and less sterically hindered compound [86]. A similar activity was achieved for Ag(I) species with nhc ligands in the case of *E. coli* and *C. albicans* biofilms [87].

On the other hand, the coordination polymer [{Cu(muco)(bpa)(2H_2_O)}·2H_2_O]_n_ (**19**) (bpa: 1,2-bis(4-pyridyl)ethane, H_2_muco: *trans*, *trans*-muconic acid) inhibited *S. aureus* biofilm formation. Furthermore, the inflammatory response of the spine surgery incision was supressed by this species [88].

Complex Cu(phen)_2_(OH_2_)](ClO_4_)_2_ (**20**) (phen: 1,10-phenantroline) proved to be a very potent antibacterial agent against both susceptible or resistant Gram-positive (*S. aureus*) and Gram negative (*E. coli*, *P. aeruginosa*) strains in planktonic or biofilm growth states. The minimum biofilm eradication concentration (MBEC) values followed a similar trend with that of MIC recorded for most of the tested strains [89]. Moreover, complexes with mixed ligands [Cu(bpy/phen)_2_(pmtp)](ClO_4_)_2_ (**21**/**22**) (bpy: 2,2′-bipyridine) showed antibacterial potential against several bacterial strains, including MRSA, extended-spectrum beta-lactamase (ESBL) producing *E. coli* and multi-drug-resistant *P. aeruginosa*, both in planktonic and biofilm growth states. In addition, both compounds exhibited superoxide scavenging ability, intercalative DNA properties, metallonuclease activity and an antiproliferative effect on B16 cells [90].

Complexes of type [M(fphen)(dach)]X_2_ (**23**) (M: Cu, Pt, Pd, fphen: functionalized 1,10-phenanthrolines, dach: 1S,2S- or 1R,2R-diaminocyclohexane, X: Cl, ClO_4_) showed significant activity against biofilms associated with a MRSA clinical isolate and were more active in the biofilm removal than vancomycin, an antibiotic currently used in the treatment of MRSA infections. The dach have no influence on activity and Cu(II) complexes, which were more active comparing with Pt(II) and Pd(II) ones as a result of nuclease activity characteristic for Cu(II) complexes bearing phen derivatives [91].

In addition to the planktonic growth inhibition, DNA binding and lack of hemolytic activity as well as significant anti-MRSA and *P. aeruginosa* biofilm activities were exhibited by the complex [Ag(mmphi)_2_]NO_3_ (**24**) (mmphi: (*E*)-7-(4-methoxybenzylidene)-3-(4-methoxyphenyl)-2-pyridyl-3,3a,4,5,6,7-hexahydro-2H-indazole) at MIC concentrations in a dose-dependent manner for *P. aeruginosa*, whereas a biphasic response was obtained for MRSA showing that the sub-MIC doses enhanced biofilm formation while its reduction was recorded at higher concentration [92].

Good activity was demonstrated for a series of complexes of type [Mn(snh)_2_X_2_] (**25**) (snh: substituted nitrogen heterocycle like pyridine or imidazole substituted with HO, CHO, CO or COOH groups, X: Cl, NO_3_, ½SO_4_) in *P. aeruginosa* biofilm eradication. The structure-activity relationship analysis evidenced an enhanced activity for pyridine derived ligands for hydroxyl as a substituent and nitrate as a counterion. In addition, complexes are non-toxic on primary human fibroblasts, exhibit catalase like activity and the ability to easily reach at Mn(III), associated with the compound’s ability to interact with biological target involved in biofilm destruction through redox processes [93].

Compounds [Ag(phendione)_2_]ClO_4_ (**26**) and [Cu(phendione)_3_](ClO_4_)_2_·4H_2_O (**27**) (phendione: 1,10-phenanthroline-5,6-dione) were tested on several carbapenemase-producing *Acinetobacter baumannii* strains, a microorganism often exhibiting a multidrug-, extended drug- and even pan-drug-resistance profile. Both compounds affected the biofilm formation and disrupted the mature biofilm sub-MIC concentration in a typically dose-dependent manner, reducing biomass and viability parameters, with Cu(II) species again being more potent [94].

The complex with mixed ligands [Zn_2_(bmic)_2_(tet)_2_]·3DMF (**28**) (H_2_bmic: 1-benzimidazole-5-carboxylic acid, tet: tetrazole DMF: N,N-dimethylformamide) was proved as a good inhibitor of *S. aureus* biofilm formation including in a bacterial infection model confirmed by counting colony forming unit (CFU) numbers in the skin following experimental infection in vivo [95].

A complex [Co(phtt)_2_]BF_4_ (**29**) (Hphtt: (*E*)-2-(2-(pyridin-2-ylmethylene)hydrazinyl)-4-(p-tolyl)thiazole) was reported as *P. aeruginosa* anti-biofilm species with a mechanism involving the transcriptional activator protein complex 3-oxo-C12-HSL-dependent QS system (LasI/LasR system) [96]. As a QS inhibitor acts also [Co(btmpp)(NCSe)_2_] (**30**) (btmpp: 2,6-bis(3,4,5-trimethylpyrazolyl)pyridine), where Co(II) adopts an unusual distorted square pyramidal geometry according with single-crystal X-ray diffraction data. The complex was screened for antibacterial activities against Gram-positive (*B. substilis*) and Gram-negative (*P. aeruginosa*, *S. typhimurium*, *Shigella sonnei* and *Y. enterocolitica*) bacterial strains. The effects of these complexes on QS-regulated behaviours of bacteria such as swarming and biofilm formation were also examined [97].

Good anti-biofilm activity was demonstrated by complex [Cu(dfx)(py)] (**31**) (dfx: deferasirox, py: pyridine) against *S. aureus* and *P. aeruginosa* together with a strong antioxidant activity [98].

By combining the relative low toxicity of essential ions (Co(II), Ni(II), Cu(II), Zn(II)) with the biological activity of N-heterocycles (pyridine, pyrimidine, imidazole and pyrazole derivatives), several valuable anti-biofilm species were developed. One of the potential mechanisms of action revealed by different studies is ROS generation.

The antimicrobial activity is enhanced when two or more isolated or fused N-heterocycle rings are present in different ligand molecules. Moreover, complexes bearing mixed ligands, both from this class of derivatives, proved to be more active compared with those having only one type of N-heterocycle in their structure.

It is worthy of mentioning the wide spectrum of most of Cu(II) complexes as well as the good activity against resistant strains such as MRSA, ESBL *E. coli* and multi-drug-resistant *P. aeruginosa*.

### 3.3. Complexes with Schiff Bases

In recent years, researchers have drawn significant attention toward Schiff bases and their metal complexes considering their numerous applications in the biological field, such as their antiviral, antimicrobial, antimalarial, and antitumor properties. Furthermore, some of complexes exhibit a good anti-biofilm activity besides the antimicrobial one against planktonic bacteria.

A series of complexes with Schiff bases bearing 1,2,4-triazole moiety of type [M(BS1)(X)]·nH_2_O (**32**) (M: Co, Ni, Cu, Zn; HBS1: 2-[(E)-(1H-1,2,4-triazol-3-ylimino)methyl phenol, X: Cl [99], CH_3_COO [100], ClO_4_ [101]) behave as good antimicrobials against both planktonic or adherent cells of a plethora of pathogenic microorganisms (*E. coli*, *K. pneumoniae*, *S. aureus*, *B. subtilis*, *C. albicans*) both on susceptible and resistant strains. The best activity was achieved for Cu(II) and Zn(II) species and, moreover, Cu(II) complexes of the series also exhibited a promising antiproliferative activity on human laryngeal carcinoma (HEp 2) and HT 29 cell lines.

Complexes [M(BS2)_2_(OH_2_)_n_] (**33**) (M: Co(II), Ni(II), Cu(II), HBS2: Schiff bases derived from cefotaxime/ceftazidime and salicylaldehyde, *n* = 2, 0) were studied as anti-biofilm species against *E. coli*, *K. pneumoniae*, *S. aureus*, and *B. subtilis*, the most active being Cu(II) compounds against *E. coli* and *P. aeruginosa* biofilms at sub-MIC concentrations [102,103]. The compounds [Ca(HBS3)(OH_2_)_4_]Cl_2_·4H_2_O (**34**) and [Cu{Ca(BS3)(OH_2_)_2_}_2_]Cl_4_·H_2_O (**35**) (HBS3: 2-hydroxy-8-methyl-tricyclo[7.3.1.02.7]tridec-13-*N*-4′(benzo-15-crown-5-ether)-imine) exhibited superior anti-biofilm activity compared to that of the ligand against several bacterial strains and *C. albicans* [104].

Anti-biofilm studies performed on *S. aureus*, *B. subtilis*, *P. aeruginosa*, *E. coli*, and *C. albicans* strains evidenced good activity at sub-MIC concentrations for [Cu(BS4/BS5)(CH_3_COO)_2_] (**36**/**37**) (BS4: Schiff bases resulted in condensation of 8-alkyl-2-hydroxy-tricyclo[7.3.1.02.7]-tridecan-13-one and 4-amino-2,3-dimethyl-1-phe nyl-3-pyrazolin-5-one, alchil: C_2_H_5_, n-C_3_H_7_, i-C_3_H_7_, C_6_H_5_; BS5: isonicotinic acid 2-(2-hydroxy-8-substituted-tricyclo[7.3.1.02.7]tridec-13-ylidene)-hydrazones, alchil: CH_3_, C_2_H_5_, n-C_3_H_7_, i-C_3_H_7_). All complexes also exhibited a relatively high toxicity on human immortalized keratinocyte (HaCaT) cells at high concentrations [105,106].

The fully characterized complexes [Cu(BS5/BS6)2] (**38/39**) (HBS5: 2-(((2,6-diisopropylphenyl)imino)methyl)-6-methylphenol; HBS6: 2-[((2,6-diisopropylphenyl)imino)methyl)-6-methoxyphenol) proved to be good inhibitors of *E. coli* biofilms at sub-MICs concentrations, activity coupled with DNA cleavage through ROS generation [107].

Compounds with the mixed ligands [Cu(BS7)(phen)] (**40**) (H_2_BS7: 3-methoxy-2-oxidobenzylidenebenzohydrazide) were characterised by single crystal X-ray diffraction, and it was found that it repressed both *P. aeruginosa* and *S. aureus* biofilm formation. Moreover, the compound exhibited the ability to intercalate into the DNA strands [108].

Overall, the available data indicate that several Cu(II) complexes with multidentate Schiff bases having N,O or N,O,O donor sets and bulky substituents like phenyl, pyridine, triazole or pyrazole exhibit a very good anti-biofilm activity on a wide range of Gram-positive and Gram-negative bacteria. The activity is enhanced for the species bearing besides Schiff base another chelate ligand such as 1,10-phenantroline. However, the specific mechanisms of action for this type of complex remains to be elucidated in future research.

### 3.4. Complexes with Biguanide Derivatives

From the perspective of anti-biofilm activity, the complexes with biguanide derivatives have also shown promising potential. The biguanides are valuable ligands that can coordinate in neutral, anionic or cationic form. Due to their chelate coordination through the imide groups they form stable complexes with transition metal ions as neutral or anionic species [109].

The metformin (*N,N*′-dimethylbiguanide, Hdmbg) compound used for type II diabetes treatment by decreasing the glucose release from the hepatic tissue is also the best known of these derivatives as complex formatters [110]. Furthermore, the dmbg moiety incorporated into a polymeric material was used as an efficient catheter coating that prevented the development of *S. aureus* and *E. coli* biofilms [111], while a novel nano-system based on a polybiguanide moiety was recently developed as a biocompatible and effective inhibitor of MRSA biofilms both in vitro and in vivo [112].

This anti-biofilm potential of biguanides motivated the research for the design of complexes with such ligands. Among these, complexes [M(Hdmbg)_2_]X_2_ (**41**) (M: Mn(II), Ni(II), Cu(II), Zn(II); X: CH_3_COO [113], and ClO_4_ [114]) with this ligand demonstrated the ability to inhibit *S. aureus* and *P aeruginosa* biofilm development on inert substratum, the most active being Cu(II) and Zn(II) complexes. Moreover, all complexes exhibited very low cytotoxicity levels on human cervical cancer (HeLa) cells.

The compounds [Fe(dmbg)_2_]Cl·0.5H_2_O (**42**) [115] and [Pd(Hdmbg)_2_]Cl_2_·H_2_O (**43**) [110] proved to exhibit an inhibitory effect on the adherence ability of *S. aureus*, *B. subtilis* and *E. coli* at low concentrations, while Pd(II) complex proved anti-biofilm activity against *C. albicans* as well [116].

Good activity against *S. aureus* and *P. aeruginosa* biofilms was demonstrated for [Cu(Htbg)_2_]Cl_2_ (**44**) (Htbg: 1-(*o*-tolyl)biguanide) [117], while the species cis-[Cu(Htbg)_2_](ClO_4_)_2_ (**45**) exhibited excellent antibacterial properties against biofilm embedded Gram-negative (*K. pneumoniae*, *Enterobacter cloacae*) and Gram-positive (*B. subtilis*, *L. monocytogenes*) bacteria, more intense in comparison with ampicillin. The best efficacy was noticed against *E. cloacae* and *L. monocytogenes* with MBEC of 1.95 µg/mL. One of the possible mechanisms of antimicrobial activity is represented by ROS generation. The complex was not cytotoxic on L929 fibroblasts, and the in silico analysis confirmed its drug-likeness and safety profile [118].

The compounds [M(Htbg)_2_]Cl_2_ (**46**) (M: Ni, Pd, Pt) proved to have good efficiency against the biofilm embedded *S. aureus*, *B. subtilis* and *E. coli* cells at sub-MIC values. The most efficient compounds showing the largest spectrum of anti-biofilm activity were Pd(II) and Pt(II) complexes. Moreover, the Pt(II) compound exhibited the most significant antiproliferative activity on the human cervical cancer (SiHa) cell line, inducing a cell cycle arrest in the G2/M phase [119].

As for complexes with mixed ligands, [M(Htbg)(Hkg)]X (**47**) (M: Ni, Cu; H_2_kg: α-ketoglutaric acid; X: Cl, CH_3_COO, NO_3_) exhibited good activity against *S. aureus* and *E. coli*, both in the planktonic and biofilm growth states [120,121].

Also, several iridium(III) species with both biguanide derivatives (bigR) and substituted cyclopentadienyl (cpR1) [Ir(bigR)(cyR1)X] (**48**) (R = Ph, 4-F-Ph, PhEt, 1-(*o*-tolyl); R1= biph) proved to be active against a large spectrum of microbial strains, including MRSA, being able to also disrupt and eradicate the bacterial mature biofilm [122].

Recent studies indicated that the biguanide incorporation into a macrocycle ligand generated promising complexes [M(dmbgMc)] (**49**) (M: Ni, Cu; H_2_dmbgMc: ligand resulted from Hdmbg condensation with ammonia/hydrazine and formaldehyde) for applications in the treatment of infections produced by pathogenic microorganisms, including those complicated by biofilm development. A broad anti-biofilm spectrum on *P. aeruginosa*, *E. faecium*, *E. faecalis*, *C. albicans* and *C. parapsilosis* was registered for Ni(II) complex with macrocycle resulted in ammonia system [123].

A good anti-biofilm activity was obtained by combining the biguanides ability to disrupt biofilm with their chelate properties. These generate several valuable species with both essential (Cu(II), Zn(II)) and non-essential (Ni(II), Pd(II), Pt(II), Ir(III)) metal ions. The anti-biofilm activity is enhanced for perchlorate species and biguanides bearing bulky substituents as a result of the favourable balance between their water solubility and lipophilicity. The ROS generation was reported as one of the potential mechanisms of action for Cu(II) species.

### 3.5. Complexes with Macrocyclic Ligands

A series of complexes with macrocycle (mc) ligands [M(mc1/mc2)Cl_2_]·nH_2_O (**50/51**) (M: Ni, Cu, Zn; mc1: 1,3,5,8,11-pentaazacyclotridecane-3-yl-(pyrid-3-yl)-methanone; mc2: (4,5,11,12)-bisphenylen-1,3,6,8,10,13-hexaazacyclotetradecan-bis(pyrid-3-yl)methanone) synthesized by the template condensation were screened for anti-biofilm activity on both susceptible and resistant strains of *P. aeruginosa*, *E. coli*, *K. pneumoniae*, *S. aureus*, *B. subtilis* and *C. albicans*, and proved to be strong inhibitors in most cases at sub-MIC concentrations, especially the Cu(II) species. These species also exhibited antiproliferative activity on HEp 2 cells by inducing the cellular cycle arrest in the G2/M phase [124,125].

The complex [Cu(mc3)]Cl_2_ (**52**) (mc3: macrocycle synthesised by the condensation reaction between substituted carbohydrazone and thiosemicarbazide) was found to be able to disrupt the biofilm produced by MRSA [126] while the antibiofilm activity of EDTA-based phenylene macrocycle (edtaod) on *L. monocytogenes*, *P. aeruginosa*, *S. typhimurium* and *S. aureus* was enhanced by its complexation with Cu(II) and Fe(III), activity that is similar and closely related with the molecular volume of EDTA complexes [127].

Moreover, a series of complexes with bismacrocycle (bmc) ligands [M_2_(bmc1)](CH_3_COO)_4_ nH_2_O (**53**) (M: Ni, Cu, Zn; bmc1: 1,2-bis(*N*,*N*-1,3,6,9,12-pentaazacyclotridecane)-benzene) were designed as antimicrobials and Zn(II) complex exhibit ability to inhibit the *S. aureus*, *B. subtilis*, *E. faecalis*, *K. pneumoniae*, *E. coli*, *E. cloacae*, *P. aeruginosa* and *C. krusei* adherence on inert substratum [128].

A similar multi-component reaction involving metal ion, amines and formaldehyde has been used for another series of decaaza bismacrocycle-based complexes [M_2_(bmc2)X_4_·nH_2_O (**54/55**) (M: Ni, Cu, Zn, bmc2: 1,3-bis(*N*,*N*-1,3,6,9,12-pentaazacyclotridecane)-benzene, X: Cl [129], CH_3_COO [130]) development as anti-biofilm species. The assays on a plethora of microorganisms (*B. subtilis*, *E. faecalis*, *S. aureus*, *S. epidermidis*, *E. cloacae*, *E. coli*, *K. pneumoniae*, *Proteus mirabilis*, *P. aeruginosa*, *C. albicans* and *C. krusei*) demonstrated good anti-biofilm activity of Cu(II) complexes on fungal strains, the acetate species being more active compared with the chloride one. Moreover, complexes are not cytotoxic on the HCT 8 tumour cells.

On the other hand, compounds of type [M_2_(bmc3)Cl_4_]·nH_2_O (**56**) (M: Ni, Cu, Zn; bmc3: 1,4-bis(*N*,*N*-1,3,6,9,12-pentaazacyclotridecane)-benzene) exhibited good anti-biofilm activity, especially against *S. aureus* and *E. coli* strains, the most active being the copper(II) complex. In addition, all complexes disrupt the membrane integrity of human ileocecal adenocarcinoma (HCT 8) tumour cells [131].

Some bio-efficient macrocycle complexes, [M_2_(bmc4)Cl_4_]·nH_2_O (**57**), (M: Co, Ni, Cu, Zn; bmc4: 1,1′-biphenyl-bis(1,6,9,14-tetrahydro-3,4;11,12-diphenyl-1,6,9,14-tetraazacyclo hexadecane were also designed as biofilm inhibitors on *S. aureus*, and among them, the Cu(II) complex displayed good potential [132].

These reports evidenced a good anti-biofilm activity for both mono- and bisazamacrocycle complexes of Ni(II), Cu(II) and Zn(II) against a wide range of Gram-negative and Gram-positive bacterial strains. However, future studies are needed to explore their specific mechanisms of action.

### 3.6. Complexes with Miscellaneous Ligands

A good inhibition against *S. marcescens* and *C. albicans* biofilm was evidenced for polymeric complex (H_2_apa)_2_[Mn(C_2_O_4_)_2_]·H_2_O (**58**) (Hapa: 2-aminopyridine-4-carboxylic acid) based on an oxalate linker associated with a lysozyme activity of the compound [133], while (Hmbzim)_3_[Fe(C_2_O_4_)_3_].3H_2_O (mbzim: 5-methylbenzimidazole) disrupted the biofilm only in case of *C. albicans* [134].

The metal-organic framework (MOF) {[Co_2_(bptc)(DMF)_2_(H_2_O)]DMF·H_2_O}n (**59**) (H_4_bptc: 3,3,5,5-biphenyltetracarboxylate) was prepared and structurally characterized. The results of the violet crystal staining experiment showed that the new compound significantly inhibited the formation of the *S. aureus* biofilm in vitro [135].

The complex [Cu(cur)_2_] (**60**) (cur: curcumin) inhibited biofilm formation in the case of *S. aureus* and significantly repressed the expression of lasI and lasR genes, demonstrating its QS inhibitory effect [136].

The assays performed on *E. coli*, *P. multocida* and *S. aureus* with complex [Ni(tea)_2_]·2bza (**61**) (tea: triethanolamine, Hbza: benzoic acid) indicated moderate to very good anti-biofilm activity [137].

The Cu(II) complexes of 1/2/3-(bromophenyl)-3-(1,7,8,9-tetramethyl-3,5-dioxo-4-azatricyclo[5.2.1.02,6]dec-8-en-4-yl)thiourea derivatives exhibited good biofilm inhibitory activity on *S. epidermidis* [138], while [Cu_2_(S-et/bu-thiosal)_4_(H_2_O)_2_] (**62/63**) (S-et-thiosal: S-ethyl derivative of thiosalicylic acid; S-bu-thiosal: S-butyl derivative of thiosalicylic acid) exhibited anti-biofilm activity on a clinical *S. aureus* isolate similarly or even better than doxycycline used as positive control [139]. A similar activity was evidenced for Cu(II) complexes with 3-(trifluoromethyl)phenylthiourea derivatives, activity related to the inhibition of DNA gyrase and topoisomerase IV from *S. aureus* [140].

The compound [Co(edtp)Cl)](NO_3_)·H_2_O (**64**) (edtp: *N*,*N*,*N*’,*N*’-tetrakis(2-hydroxypropyl)ethylenediamine) exhibited moderate antithrombolytic activity and negligible cytotoxicity against bovine erythrocytes and in addition a very good bacterial biofilm inhibition (90%) against both *B. subtilis* and *E. coli* strains [141].

Moreover, the complex with mixed ligands [Zn(tsa)(tmeda)]_2_ (**65**) (Htsa: thiosalicylic acid; tmeda: *N*,*N*,*N*′,*N*′-tetramethylethylenediamine) is very active on the old biofilms of *S. aureus*, as indicated in the studies performed by confocal laser scanning microscopy which revealed its bactericidal activities, possibly by membrane alterations, as demonstrated by the propidium iodide (PI) uptake [142].

Complexes [Zn(bedtcm/imdtcm)_2_] (**66/67**) (bedtcm: *N*-(benzyl)-(ethyl)-dithio carbamate, imdtcm: *N*-(4-isopropyl-benzyl)-(4-methoxy-benzyl)-dithiocarbamate) exhibited anti-biofilm activity against both methicillin susceptible and resistant *S. aureus* [143], while [Ag(aptes)_2_NO_3_] (**68**) (aptes: 3-aminopropyltriethoxysilane) showed pronounced antibacterial effects against *P. mirabilis* isolated from patients with urinary tract infections, and exhibited a clear decrease in the ability of this bacteria to form biofilms at MIC concentration [144].

There are few reports concerning the anti-biofilm activity of some multidentate ligands bearing carboxylate/thiocarboxylate, hydroxy or amino groups. The good activity reported for species bearing sulfur as donor atoms opens promising leads that will likely boost future research in the field.

**Figure 2:**
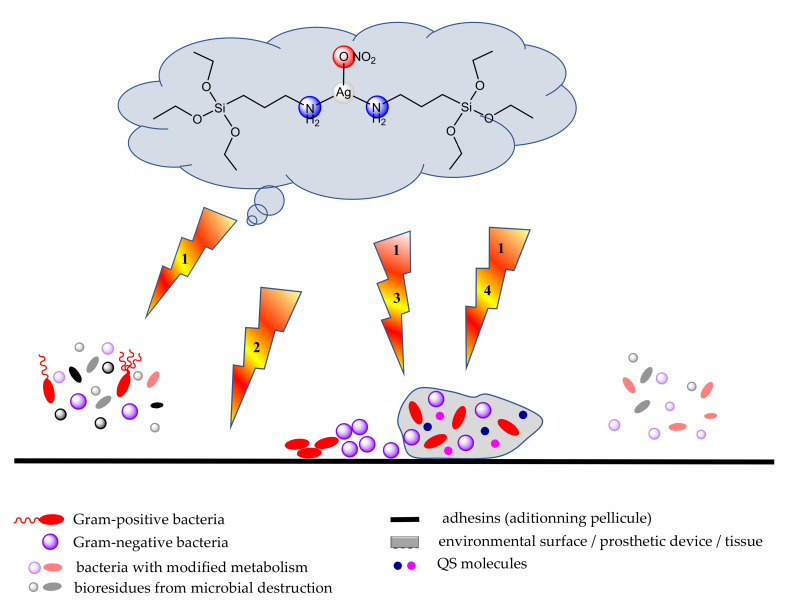
Anti-biofilm activity of complexes: 1-antimicrobial activity, 2-adherence inhibition, 3-QS inhibition and 4-biofilm destruction through ROS or NOS generation.

The anti-biofilm activity of complexes together with identified mechanisms of action is presented in Figure 2.

### 3.7. Materials as Carriers for Metal Ions or Complexes with Anti-Biofilm Activity

In order to overcome the problems associated with the use of antibiotics, some polymer species complexed with proper metal ions or loaded with biological active complexes as well as nanomaterials with anti-biofilm properties and biocompatibility/environmental safety have also been developed. Several dendrimers and polymers appropriately modified with coordinative groups able to chelate metal ions were designed for this purpose. Moreover, several attempts were made for the complexes’ incorporation into organic or inorganic matrices.

Dendrimers are branched three-dimensional macromolecules based on a nitrogen, phosphorus and silicone backbone, and carry groups able to coordinate metallic ions, properties that afford applications as metal ion carriers for anti-biofilm purposes. A proper selection of dendritic scaffold, generation type and nature of donor atom can provide a potent system that can overcome the limitations of traditional therapies with antibiotics [147]. As result, a second-generation poly(propylene imine) dendrimer modified with acridine and loaded with Cu(II) was developed first as an antimicrobial with low cytotoxicity against the human epithelial type 2 (HEp-2) cell line. Afterwards, a cotton fabric modified with this dendrimer was proved to exhibit anti-biofilm activity against *B. cereus* and *P. aeruginosa* strains, and no cytotoxicity on the HEp-2 cell line [148].

Another dendrimer from first generation of polyamidoamine (PAMAM) functionalised with 1,8-naphthalimide moiety was loaded with Cu(II) and attached to the cotton surface. The study showed that this material prevented the biofilm formation in the case of *B. subtilis*, *B. cereus* and *A. johnsonii*, the best effect being observed for the last strain [149]. Furthermore, a material based on a second generation PAMAM dendrimer modified with 4-(*N*,*N*-dimethylaminoethyloxy)-1,8-naphthalimide and conjugated with cis-Cu(NO_3_)_2_ moiety was developed and deposited on cotton fabric. The obtained composite exhibits inhibitory activity against *B. cereus*, *P. aeruginosa* and *C. lipolytica* biofilms [150]. On the other hand, a water-soluble carbosilane dendrimer, decorated with iminopyridine groups and conjugated with Cu(OH_2_)(ONO_2_)_2_ moiety (**69**), was developed as a potent species against both planktonic and biofilm embedded *S. aureus* and *E. coli* cells [151].

Among the polymeric composites, those generated by [Cu(dttct)](CH_3_COO)_2_ (**70**) (dttct: dibenzo[e,k]-2,3,8,9-tetraphenyl-1,4,7,10-tetraaza-cyclododeca-1,3,7,9-tetraene) incorporation into poly(vinyl chloride) (PVC) exhibited the ability to generate nitric oxide by nitrite reduction, a process assisted by the ascorbic acid. It was observed that this composite controlled both the formation and dispersion of nitrifying bacteria biofilm [152]. Moreover, the system obtained by complex (**70**) immobilisation together with [Fedttct]Cl_3_ (**71**) into the same polymeric matrix exhibited the same ability to generate nitric oxide (NO) from endogenous nitrite. This material exerted anti-biofilm activity against *Bacillus* sp., in addition to nitrifying bacteria biofilm dispersion [153].

A series of 2,6-pyridinedicarboxylate-based polyesters employing several diols with different aliphatic chains were synthesised and complexed with Cu(II) and Ag(I). The composites were tested for their antibacterial potential and were found to effectively resist *P. aeruginosa* attachment and colonization, the silver-based polymers being superior in comparison with their copper analogues [154].

Schiff-base ligands were grafted on a natural biopolymer of ε-poly-*L*-lysine functionalized mesoporous silica SBA-15 for the selective coordination of Ag(I). This nano-species (**72**) exhibited inhibitory effect on *E. coli*, *S. aureus*, *M. tuberculosis* and *C. albicans*. Besides killing the *C. albicans* cells, this system inhibited biofilm formation and eliminated preformed biofilms, with no development of resistance during continuous serial passaging. The antifungal activity is related to the disruption of bacterial cell membranes and increased levels of intracellular ROS. In mouse models of multidrug-resistant *C. albicans* infection, nano-species exhibited an efficient in vivo fungicidal efficacy superior to the antifungal drugs, amphotericin B and fluconazole. Moreover, treatment induced negligible toxicity against normal tissues [155].

Anti-biofilm agents based on Ga(III) or zinc Zn(II) complexed with protoporphyrin IX or mesoprotoporphyrin IX were found to be highly effective in the inhibition of both planktonic bacterial growth and biofilm formation. These complexes were incorporated in poly(ether urethane) (PEU) polymer films in order to obtain a system for their controlled sustained release by using poly(ethylene glycol) (PEG) as a pore-forming agent. All complex-loaded PEU films exhibited in vitro a ≥ 90% reduction of *S. epidermidis* and *P. aeruginosa* in both suspended and biofilm culture. Moreover, the cytotoxicity and endotoxin evaluation demonstrated no adverse responses, while in vivo studies further substantiated the anti-biofilm efficacy of these composites [156].

A composite material based on polylactic acid (PLA) fibres containing cobalt-based MOF [Co(mcim)_4_](NO_3_)_2_ (**73**) (mcim: 4-methyl-5-carboxyaldehyde-imidazole) was prepared by electrospinning PLA with a suspension of polyvinylpyrrolidone-stabilized of (**73**). MOF particles formed aggregates that, after being electrospun, became completely embedded inside of polymeric fibres, which inhibited t *S. aureus* biofilm [157].

Another material based on water-stable MOF H_3_[(Cu_4_Cl)_3_(BTTri)_8_] (H_3_BTTri: 1,3,5-tris(1*H*-1,2,3-triazol-5-yl)benzene) blended with chitosan demonstrated the ability to reduce by 85% the *P. aeruginosa* adhesion in the first 6 h, and maintained the inhibitory effect up to 24 h. Moreover, the system elicited the same inhibitory effect after a second round of experiments, suggesting reusability of the materials [158].

The development of an effective treatment for MRSA infections is complicated by the fact that antibiotics can be degraded by β-lactamases, and the antibiotics cannot penetrate the full depth of biofilms. Considering the nanoparticle-based carriers’ ability to deliver antibiotics with better biofilm penetration, a platform for β-lactam antibiotics and β-lactamase inhibitors co-delivery based on metalcarbenicillin framework-coated mesoporous silica nanoparticles (MSN) was developed. Carbenicillin, a β-lactam antibiotic, was used as a ligand for Fe(III) in order to generate a metalcarbenicillin framework able to block the pores of the MSN. The study evidenced that this system achieved a better penetration in the depth of biofilms and exhibited an inhibitory effect on the MRSA biofilm both in vitro and in vivo [159].

Despite the current tendency to use drug delivery systems (DDSs) based on biocompatible and biodegradable matrices, the studies concerning the use of DDS for anti-biofilm species are rather few. The available studies are reporting Cu(II) or Ag(I) coordination to dendrimers or natural or synthetic polymers providing N as donor atoms or the incorporation of some complexes into polymeric (linear or branched) or silica matrices, or even in organic-inorganic composites.

## 4. Conclusions

Biofilm development on viable tissues and prosthetic devices represents an important challenge for the medical field, due to their involvement in biofilm-associated infections, which are persistent and hard or often impossible to treat. Therefore, finding efficient agents capable of surpassing the numerous mechanisms of biofilms tolerance to high doses of antimicrobials represents one of the hot fields of research for both microbiologists and chemists. Metal complexes offer promising leads for the development of biofilm disrupting agents due to their multi-target, complex mechanism of action. The current data reveal the efficiency of metal complexes against biofilms formed by epidemiologically important resistant strains, such as MRSA. From the point of view of ligands, it was observed that nitrogen-based ligands mostly involved in chelation lead to an enhanced anti-biofilm activity, and Cu(II) complexes with these species exhibit the most promising activity associated with biofilm disruption. However, most studies in the field are focused only on assessing the in vitro anti-biofilm activity, and very few address the elucidation of the intimate mechanisms of action; the current studies identified the QS inhibition or ROS/NOS generation as some of the main mechanisms involved in biofilm disruption by metal complexes. From these data it is obvious that this represents an open field, and there are many aspects that must be elucidated by further studies.

One of the most promising leads for the design of new complexes with anti-biofilm activity are the redox active metal ions such Cu(II), Fe(III) and Mn(II) but the less-studied ones such VO(IV) and Ru(II) should be also considered. All of these ions have ROS or NOS generation as a common mechanism of action. The best anti-biofilm activity is achieved then these ions are combined with multidentate ligands, especially bearing N as donor atoms, assuring enhanced stability. Furthermore, the perchlorate anion that easily generates single crystals seems to enhance the anti-biofilm activity in complexes bearing neutral organic ligands. The most active compounds show an improved activity after incorporation in organic, inorganic or composite matrices. The majority of the current literature refers to the in vitro study of the anti-biofilm activity of complexes, this explaining the paucity of novel anti-biofilm agents in medical practice. Thus, there is an urgent need for additional in vivo studies in this field in order to elucidate the safety, efficacy and toxicity of these species in order to develop new valuable drugs for the treatment of biofilm-associated infections.

## Figures and Tables

**Table 1:** Examples of complexes with anti-biofilm activity.

Complex	Metallic Ion	Microorganism/ Biofilm Assay Method	Mechanism	Ref.
**Antibiotic Ligand**				
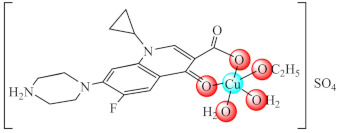 (**1·EtOH**)	Cu(II)	*P. aeruginosa*/crystal violet (CV)	QS inhibition (*lasI* and *lasR* genes modified expression)	[65,66]
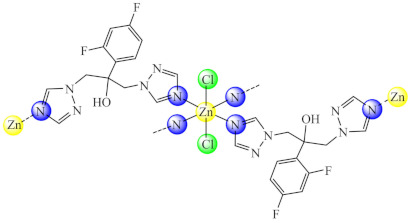 (**2**)	Cu(II), Zn(II)	*C. albicans*, *P. aeruginosa*/CV	ND	[67]
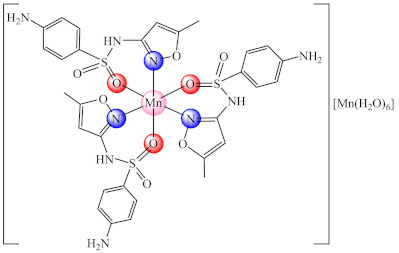 (**3**)	Mn(II)	*S. aureus*/CV	ND	[68]
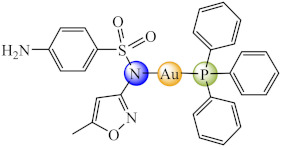 (**4**)	Au(I), Cu(II), Ag(I), Hg(II), Cd(II)	*M. abscessus*, *M. fortuitum*, *M. massiliense*/CV	ND	[70]
**N-based Heterocycle Ligand**				
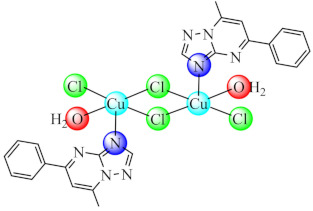 (**5**)	Co(II), Ni(II), Cu(II), Zn(II)	*E. coli*, *K. pneumoniae*, *P. aeruginosa*, *S. aureus*, *B. subtilis*/CV	ND	[74]
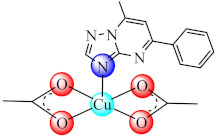 (**6**)	Co(II), Ni(II), Cu(II), Zn(II)	*E. coli*, *K. pneumoniae*, *P. aeruginosa*, *S. aureus*, *B. subtilis*, *C. albicans*/CV	ND	[75]
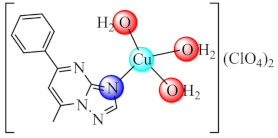 (**7**)	Co(II), Ni(II), Cu(II), Zn(II)	*E. coli*, *K. pneumoniae*, *P. aeruginosa*, *S. aureus*, *B. subtilis*, *C. albicans*/CV	ND	[76]
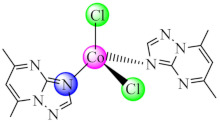 (**8**)	Co(II)	*E. coli*, *K. pneumoniae*, *P. aeruginosa*, *S. aureus*, *B. subtilis*, *C. albicans*/CV	ND	[77]
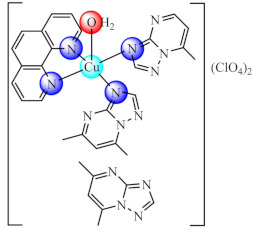 (**10**)	Cu(II)	*E. coli*, *P. aeruginosa*, *S. aureus, MRSA*/CV	ROS generation	[78]
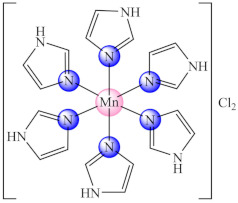 (**11**)	Mn(II)	*E. coli*/Fluorescence microscopy and XTT (2,3-bis(2-methoxy-4-nitro-5-sulfophenyl)-5-[(phenylamino)carbonyl]-2*H*-tetrazolium hydroxide)	ND	[79]
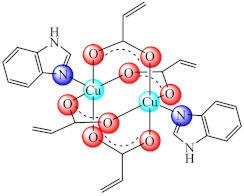 (**12**)	Cu(II)	*E. coli*, *S. aureus*, *B. subtilis*, *E. faecium*, *C. albicans*/CV	ND	[80]
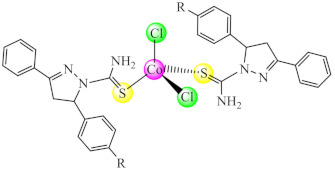 (**14**) R: Cl, Br	Co(II)	*C. glabrata*/XTT	ND	[82]
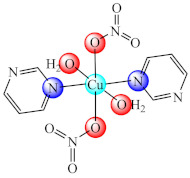 (**15**)	Cu(II)	*P. aeruginosa*/CV	QS inhibition	[83]
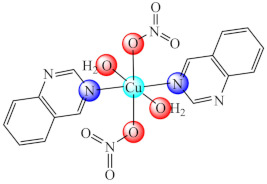 (**16**)	Cu(II)	*P. aeruginosa*/CV	QS inhibition	[83]
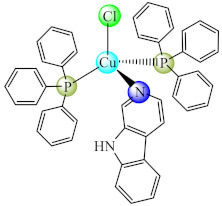 (**17**)	Cu(I)	*P. aeruginosa*, *E. coli*, *C. violaceum*, *S. marcescens*, *K. pneumoniae, L. monocytogenes*/CV	QS inhibition (violacein, elastase, pyocyanin, alginate, prodigiosin production inhibition)	[84]
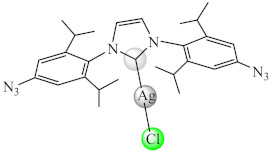 (**18**)	Cu(I), Ag(I)	*L. monocytogenes*, *S. aureus*, *S. epidermidis*, *P. aeruginosa*, *E. coli*/CV	ND	[85]
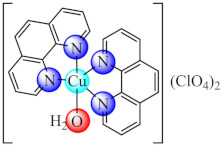 (**20**)	Cu(II)	*S. aureus*, *E. coli, P. aeruginosa*/CV	ND	[89]
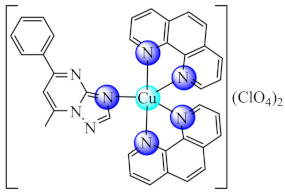 (**22**)	Cu(II)	*S. aureus*, *E. coli, P. aeruginosa*/CV	ROS generation	[91]
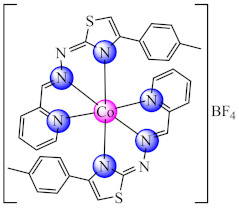 (**29**)	Co(III)	*P. aeruginosa*/CV alamar blue	QS inhibition (*lasI* and *lasR* inhibition) reduced level of pyocyanin and pyoverdine virulence factors	[96]
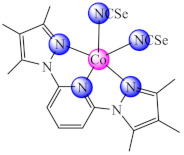 (**30**)	Co(II)	*B. substilis*, *P. aeruginosa*, *S. typhimurium*, *S. sonnei*, *Y. enterocolitica*/CV	swarming bacterial motility inhibition	[97]
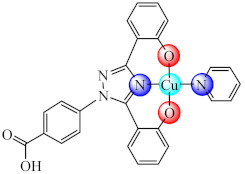 (**31**)	Cu(II)	*S. aureus*, *P. aeruginosa*/CV	ND	[98]
**Schiff Base Ligands**				
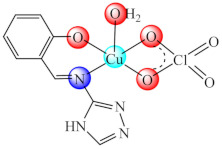 (**32**)	Co(II), Ni(II), Cu(II), Zn(II)	*E. coli*, *K. pneumoniae*, *S. aureus*, *B. subtilis*, *C. albicans*/CV	ND	[101]
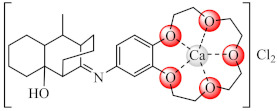 (**34**)	Ca(II), Cu(II)	*S. aureus*, *B. subtilis*, *P. aeruginosa*, *E. coli*, *C. albicans*/CV	ND	[104]
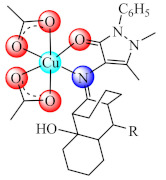 R: C_2_H_5_, n-C_3_H_7_, i-C_3_H_7_, C_6_H_5_ (**36**)	Cu(II)	*S. aureus*, *B. subtilis*, *P. aeruginosa*, *E. coli*, *C. albicans*/CV	ND	[105]
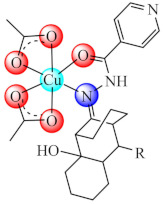 R: CH_3_, C_2_H_5_, n-C_3_H_7_, i-C_3_H_7_, (**37**)	Cu(II)	*S. aureus*, *P. aeruginosa*, *E. coli*, *C. albicans*/CV	ND	[106]
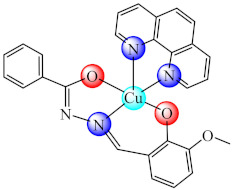 (**40**)	Cu(II)	*P. aeruginosa*, *S. aureus*/CV	ND	[108]
**Biguanide Ligands**				
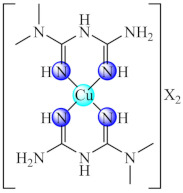 (**41**) X: CH_3_COO, ClO_4_	Mn(II), Ni(II), Cu(II), Zn(II)	*S. aureus*, *P. aeruginosa*/CV	ND	[113,114]
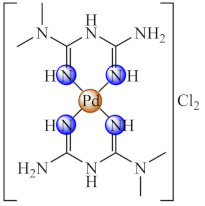 (**43**)	Pd(II)	*S. aureus*, *B. subtilis*, *E. coli*, *C. albicans*/CV	ND	[116]
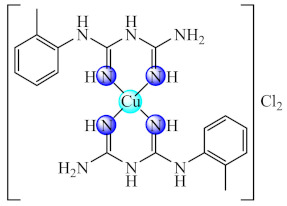 (**44**)	Cu(II)	*S. aureus*, *P. aeruginosa*/CV	ND	[117]
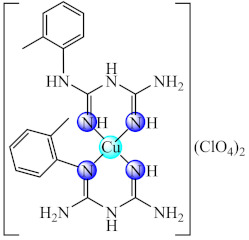 (**45**)	Cu(II)	*K. pneumoniae*, *E. cloacae*, *B. subtilis*, *L. monocytogenes*/CV	ROS generation	[118]
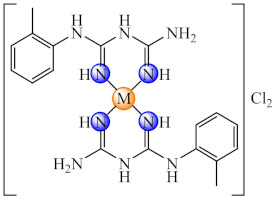 (**46**)	Ni(II), Pd(II), Pt(II)	*S. aureus*, *B. subtilis*, *E. coli*/CV	ND	[119]
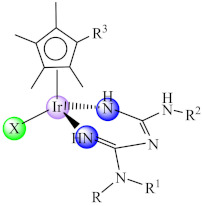 (**48**)	Ir(III)	*S. aureus*/CV	ND	[122]
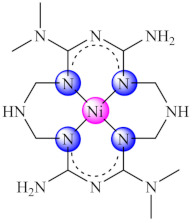 (**49**)	Ni(II), Cu(II)	*P. aeruginosa*, *E. faecium*, *E. faecalis*, *C. albicans*, *C. parapsilosis*/CV	ND	[123]
**Macrocyclic Ligands**				
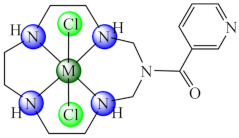 (**50**)	Ni(II), Cu(II), Zn(II)	*P. aeruginosa*, *E. coli*, *K. pneumoniae*, *S. aureus*, *B. subtilis*, *C. albicans*/CV	ND	[124]
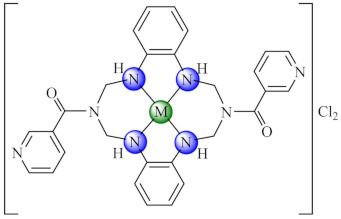 (**51**)	Ni(II), Cu(II), Zn(II)	*P. aeruginosa*, *E. coli*, *K. pneumoniae*, *S. aureus*, *B. subtilis*, *C. albicans*/CV	ND	[125]
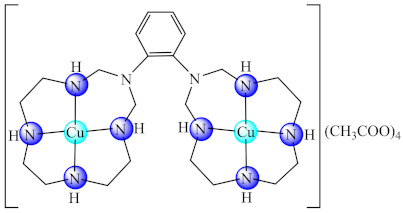 (**53**)	Ni(II), Cu(II), Zn(II)	*S. aureus*, *B. subtilis*, *E. faecalis*, *K. pneumoniae*, *E. coli*, *E. cloacae*, *P. aeruginosa*, *C. krusei*/CV	ND	[128]
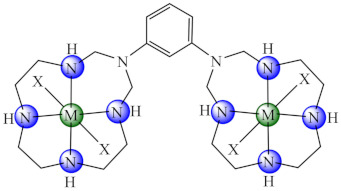 (**54**) X: Cl, (**55**) X: CH_3_COO	Ni(II), Cu(II), Zn(II)	*B. subtilis*, *E. faecalis*, *S. aureus*, *S. epidermidis*, *E. cloacae*, *E. coli*, *K. pneumoniae*, *P. mirabilis*, *P. aeruginosa*, *C. albicans*, *C. krusei*/CV	ND	[129,130]
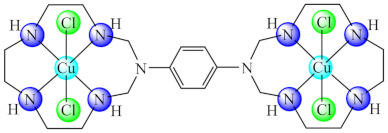 (**56**)	Ni(II), Cu(II), Zn(II)	*B. subtilis*, *E. faecalis*, *S. aureus*, *S. epidermis*, *E. cloacae*, *E. coli*, *K. pneumoniae*, *P. mirabilis*, *P. aeruginosa*, *C. albicans*, *C. krusei*/CV	ND	[131]
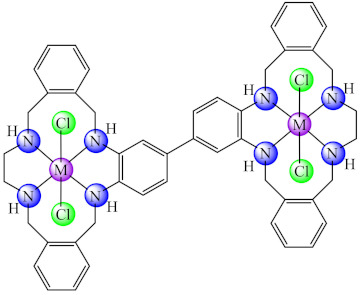 (**57**)	Co(II), Ni(II), Cu(II), Zn(II)	*S. aureus*/XTT	ND	[132]
**Miscellaneous Ligands**				
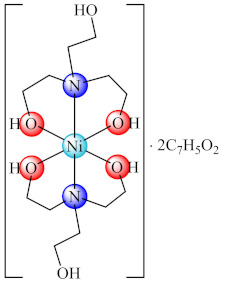 (**61**)	Ni(II)	*E. coli*, *P. multocida*, *S. aureus*/CV	ND	[137]
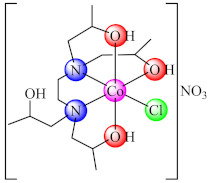 (**64**)	Co(II)	*B. subtilis*, *E. coli*/CV	ND	[141]
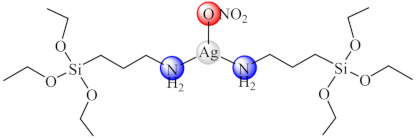 (**68**)	Ag(I)	*P. mirablis*/CV	ND	[144]
**Carriers for Metal Ions or Complexes**				
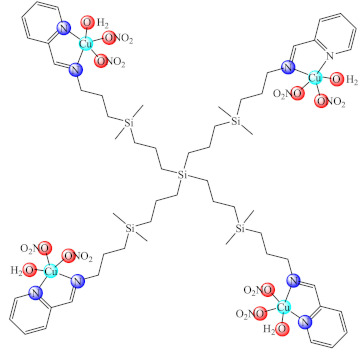 (**69**)		*S. aureus*, *E. coli*/CV	ND	[151]
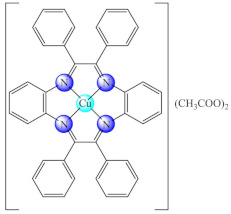 (**70**)	Cu(II)	*Bacillus* sp.		[152]
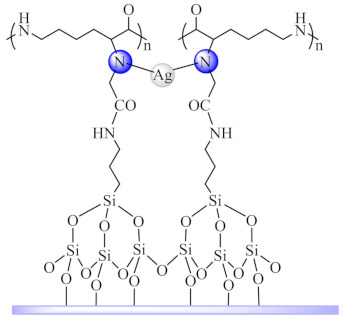 (**72**)	Ag(I)	*E. coli*, *S. aureus*, *M. tuberculosis*, *C. albicans*/XTT	NO generation	[155]

ND-not determined.

## Data Availability

Not applicable.

## Abbreviations

amx	amoxicillin
aptes	3-aminopropyltriethoxysilane
bedtcm	*N*-(benzyl)-(ethyl)-dithio carbamate
bigR	biguanide derivatives
bmc	bismacrocycle
bpa	1,2-bis(4-pyridyl)ethane
bpy	2,2′-bipyridine
btmpp	2,6-bis(3,4,5-trimethylpyrazolyl)pyridine)
cbl	β-carboline
c-di-GMP	cyclic-di-guanosine monophosphate
CFU	colony forming units
cpR	substituted cyclopentadienyl
cur	curcumin
cv	crystal violet
dach	1*S*,2*S*- or 1*R*,2*R*-diaminocyclohexane
dfx	deferasirox
DMF	*N*,*N*-dimethylformamide
dmtp	5,7-dimethyl-1,2,4-triazolo[1,5-*a*]pyrimidine
dttct	dibenzo[e,k]-2,3,8,9-tetraphenyl-1,4,7,10-tetraaza-cyclododeca-1,3,7,9-tetraene
edtaod	EDTA-based phenylene macrocycle
edtp	*N*,*N*,*N*′,*N*′-tetrakis(2-hydroxypropyl)ethylenediamine
EPS	extracellular polymeric substances
fcz	fluconazole
fphen	functionalized 1,10-phenanthrolines
H_2_bmic	1-benzimidazole-5-carboxylic acid
H_2_dmbgMc	ligand resulted from Hdmbg condensation with ammonia/hydrazine and formaldehyde
H_2_kg	α-ketoglutaric acid
H_2_muco	*trans*, *trans*-muconic acid
H_3_BTTri	1,3,5-tris(1*H*-1,2,3-triazol-5-yl)benzene
H_4_bptc	3,3,5,5-biphenyltetracarboxylate
H_4_EDTA	ethylenediaminetetraacetic acid
Hacr	acrylic acid
Hapa	2-aminopyridine-4-carboxylic acid
Hbza	benzoic acid
Hbzim	benzimidazole
Hcip	ciprofloxacin
Hdmbg	*N,N*′-dimethylbiguanide
Him	imidazole
Hphtt	(*E*)-2-(2-(pyridin-2-ylmethylene)hydrazinyl)-4-(p-tolyl)thiazole
Htbg	1-(*o*-tolyl)biguanide
Htsa	thiosalicylic acid;
imdtcm	*N*-(4-isopropyl-benzyl)-(4-methoxy-benzyl)-dithiocarbamate
mcim	4-methyl-5-carboxyaldehyde-imidazole
Me_2_bzim	5,6-dimethylbenzimidazole
MIC	minimum inhibitory concentration
mmphi	(*E*)-7-(4-methoxybenzylidene)-3-(4-methoxyphenyl)-2-pyridyl-3,3a,4,5,6,7-hexahydro-2*H*-indazole
MOF	metal-organic framework
MRSA	methicillin resistant *S. aureus*
MSN	mesoporous silica nanoparticles
nhc	*N*-heterocyclic carbene
PAMAM	polyamidoamine
PEU	poly(ether urethane)
Ph	phenyl
phen	1,10-phenantroline
phendione	1,10-phenanthroline-5,6-dione
pmtp	5-phenyl-7-methyl-1,2,4-triazolo[1,5-*a*]pyrimidine
PVC	poly(vinyl chloride)
py	pyridine
pym	pyrimidine
QS	quorum sensing
quz	quinazoline
RNS	reactive nitrogen species
ROS	reactive oxygen species
S-bu-thiosal	S-butyl derivative of thiosalicylic acid
S-et-thiosal	S-ethyl derivative of thiosalicylic acid;
smx	sulfamethoxazole
snh	substituted nitrogen heterocycle
tbpp	1-thiocarbamoyl-5-(4-bromophenyl)-3-phenyl-4,5-dihydro-1*H*-pyrazole
tcpp	1-thiocarbamoyl-5-(4-chlorophenyl/)-3-phenyl-4,5-dihydro-1*H*-pyrazole
tea	triethanolamine
tet	tetrazole
tmeda	*N*,*N*,*N*′,*N*′-tetramethylethylenediamine
XTT	2,3-bis(2-methoxy-4-nitro-5-sulfophenyl)-5-[(phenylamino)carbonyl]-2*H*-tetrazolium hydroxide
